# The *Inverted Pyramid* and Beyond: Perceptions of Distributive Justice Among (Highly) Qualified Workers in Contemporary Cuba

**DOI:** 10.1177/13607804241259279

**Published:** 2024-09-04

**Authors:** Nina Jany, Monica Budowski

**Affiliations:** University of Fribourg, Switzerland; University of Fribourg, Switzerland

**Keywords:** Cuba, distributive justice, equality, equity, multiprinciple approach of justice, need, wages, work

## Abstract

In 2020/2021, wage scale reforms were implemented in Cuba to improve distributive justice. The previously long-prevailing wage system and resulting wage structure, often referred to as an *inverted pyramid*, is said to have especially affected (highly) qualified workers in Cuba. This research provides emergent and partly speculative data on perceptions of distributive justice that (highly) qualified workers in Havana held during the time of change. The results of the exploratory study reinforce and illustrate the common claim in the literature that wage differentiation based on equity principles receives broad support. At the same time, we show how principles of need and equality also have their place in the interviewees’ reasonings. Finally, we outline two different patterns that interviewees invoke to explain why they perceive equity violations as unjust.

## Introduction

Academic, media, and political discourses about Cuban salaries often use the metaphor of an *inverted pyramid* to describe the Cuban wage system and its resulting wage structure that prevailed for the past six decades. The so-called *wage egalitarianism* in the state sector and its adverse consequences, that is (highly) qualified professionals in the state sector earning much less than low-qualified occupations in other sectors of the economy, have long been criticised – not least by the leading figures of the Cuban Communist Party (e.g. Castro, 2014). In the context of the wider transformations of the state-planned economy, the past decade has seen reforms of this wage system culminating in the so-called new monetary order in 2020/2021. As part of the currency unification,^1^ wages were not only generally raised but also (further) differentiated according to professions and qualifications. The principle ‘to each according to ability, to each according to work’ has often been mentioned in this context; it is even anchored in the current constitution (Constitución de la República de Cuba, 2018: Art. 65).

Distributive justice literature refers to the above justice principle – or at least to its second part, ‘to each according to work’ – as the *equity principle* (sometimes also *contribution*-, *achievement*-, or *proportionality* rule or principle). Various studies show that there is broad support for this principle across different social groups, countries, and cultures, especially regarding wage distribution. Early research on attitudes towards occupational earnings hierarchies and ratios found that ‘equality of earnings is nearly universally rejected by the public of industrial societies, Communist (Sarapata, 1963) as well as capitalist’ (Alves and Rossi, 1978: 547; Kelley and Evans, 1993). Quantitative studies conclude that, despite some nuances, there is at least partial consensus among countries regarding the general order of the occupational earnings hierarchies, as well as desired wage levels at the bottom of these hierarchies (e.g. Gijsberts, 2002; Kelley and Evans, 1993). The research also states that individuals and nations differ in their attitudes towards occupational hierarchies and income ratios. This is especially true for the overall level of income inequality and the income differences that people consider desirable between different occupations (e.g. Andersen and Yaish, 2012; Aristei and Perugini, 2016; Hadler, 2005; Ignácz, 2018; Kelley and Evans, 1993; Kelley and Zagorski, 2005; Osberg and Smeeding, 2006; Redmond et al., 2002). Although the general order of occupational earnings hierarchies within and across countries is less controversial, some studies point to national (or cultural) differences in this regard. This is especially the case when comparing (former) state-socialist to market-capitalist societies (e.g. Duru-Bellat and Tenret, 2012; Gijsberts, 2002), e.g. regarding the (e)valuation of blue- versus white-collar workers (Gijsberts, 2002).

Theoretically, the original formulation of equity is *vague* or *flexible* enough to be interpreted in different ways and to be compatible with other justice principles (Fischer, 2016). Advocates of a *multi-principle approach of justice* stress that different principles of justice can coexist (with equity, equality, and need usually named as overriding principles) and that finer distinctions are possible, that is, that they can be divided into sub-principles (see, e.g. Törnblom and Kazemi, 2015). For example, equity could be subdivided into the principles of productivity, effort, and ability; equality could be subdivided into the principles of equality of treatment, outcomes, and opportunity; and need could be subdivided into the principles of biological, basic, and functional need (Törnblom and Kazemi, 2015).^2^

Against the backdrop of these theoretical considerations and empirical findings on market-capitalist countries in the Western world and/or former state-socialist countries, this article focuses on perceptions of distributive justice in contemporary Cuba (Havana). The Caribbean Island has formally remained a constitutionally socialist state with a mainly state-led and centrally planned economy that has started opening its markets to private businesses for some years now. When the wage reforms described above were introduced, we interviewed (highly) qualified workers, the group that is said to be most affected by the *inverted pyramid*, about their perceptions of wages and distributive justice. The research, based on face-to-face interviews and an online survey, has a preliminary and explorative character. The results illustrate interviewees’ arguments in favour of wage differentiation based on equity principles, but also point to principles of (basic) need and equality (of outcomes). These findings tie in with the existing literature and may serve as a basis for future research, in Cuba and beyond.

## Data and methods

The analysis presented in this article draws on two data sets: face-to-face interviews (N = 19) and an online survey (N = 91), with the former being the main basis for analysis. A common characteristic among both samples is that the respondents are highly qualified workers, as determined by their initial vocational or professional degrees, hailing from various professions and occupations in Havana.^3^ This group seems to be most impacted by the inverted pyramid and is therefore likely to have thoughts about the topic. The online survey was developed in the context of the COVID-19 pandemic^4^ in collaboration with the Department of Sociology at the University of Havana and was conducted between June and September 2020. Most of the survey items were sourced from international surveys, such as the International Social Survey Programme and the International Social Justice Project, and were partially adjusted to align with the Cuban context. The survey includes questions concerning personal satisfaction (with work, salary/income, and life in general); occupational rankings; the assessment of wage criteria; (dis)agreement with statements on income inequality, redistribution, work; as well as socio-demographic questions. Initially, the questionnaire was distributed through mailing lists of the University of Havana, primarily targeting students and employees in the social sciences, economics, humanities, and natural sciences. Subsequently, it was disseminated through a snowballing approach. This has resulted in a rather homogeneous sample in terms of the participants’ educational level, with 77 out of 91 respondents reporting possession of a university degree and 14 having completed high-school education. The sample is predominantly female (56 women, 33 men, and 2 unassigned) and is relatively young (48 participants aged 21–30, 34 participants aged 31–55, and 9 participants aged 56–65). These distinctive sample characteristics offer some contextualization for insights; however, they impose limitations on the ability to draw inferences (Baur and Florian, 2009). Given these constraints, we use the data solely for descriptive purposes to complement the findings of the qualitative interview analysis.

Face-to-face interviews were conducted between November 2020 and March 2021. Interviewees were drawn either from the pool of survey participants or identified through a snowballing approach. When selecting participants, we followed a purposive sampling strategy of *least similar cases* in order to achieve heterogeneity in terms of professions, biographical trajectories, gender (9 women, 10 men), and age (ranging from 23 to 60).^5^ The interview guideline contained narrative and dialogue elements (Witzel and Reiter, 2012). Key topics covered in the interview guide included the interviewees’ work biography; supplementary questions about decisions and motivations regarding this biography (particularly concerning economic considerations, work satisfaction, reflections on social (in)justice, and the interviewees’ concept of work); an assessment of the current situation of the Cuban realm of work, its biggest problem(s) and ongoing reforms; and socio-demographic questions. Interview transcripts were analysed with grounded theory methodology, that is, using open, axial, and selective coding, as well as memos and comments (Corbin and Strauss, 2008). Essential guiding questions in the coding process were as follows: What role do distributive justice in general, and justice principles such as equity, equality, and need play in the interviewees’ lives? Do the interviewees address these principles when describing their work biography, and if so, how?

## Discussion of results

### Support for equity principles and wage differentiation

In nearly all interviews, wages are a prominent theme, particularly in response to the question regarding the biggest problem in the Cuban realm of work. Most interviewees criticise the wage system that has prevailed in Cuba over the past decades. Our bottom–up analysis reveals reasoning patterns that rely on equity principles and are connected to the idea of wage differentiation according to *qualification*. This result aligns with the literature on distributive justice. For example, when Alfredo (46), who was trained as a school teacher but has been working as a tourist guide for the past two decades, was asked about the ongoing reforms at the time of the interview, he replied:

I approve. I approve. Let’s see, eh . . . years ago when I started as a teacher it wasn’t the same salary as it is now. It wasn’t the SAME. Eh, years ago, for example, a master or a doctor . . . earned forty or fifty pesos, or a hundred pesos more, not now [now it is different]. And so, it is like they are starting to value professionals. I don’t know if I am making myself understood. And I think we needed it. A doctor, a lawyer . . . Do you understand? So, sometimes, for example, you would meet someone in the street, who worked . . . in a cab and who earned more than a doctor. Unjust! Unjust! So, I think that, that, that . . . that the pyramid is . . . taking its shape. And I like that, I like that. (Alfredo, para. 34)

Much like Alfredo and several other interviewees, Pavel (26), who dropped out of his studies in industrial engineering and stays afloat with part-time, unqualified work, portrays the wage structure in Cuba as an *inverted pyramid* and regards it as the main problem with the Cuban labour situation. Similar to his peers, he invokes equity principles to describe and explain the situation. In contrast to Alfredo, Pavel does not directly describe the perceived problems as *unjust*. When asked how the problem of the inverted pyramid could be solved, he responds:

I think to equalize salaries a bit and to give more to . . . to, to whoever has more merits. The state has done that in a way, but, for example, ME, getting paid, being an engineer, graduating in industrial engineering, which is what I was studying, I will never be able to get paid what I work as a waiter, in a private restaurant. [Interviewer: Mhm, and that seems unjust to you?] Mh, it doesn’t seem unjust to me s-, eeh, because . . . I don’t know how to explain it. In a way, I think that the government should . . . stimulate its workers more. (Pavel, para. 43)

Pavel’s difficulty in articulating his views reveals ambivalence: while he advocates for equity (referring to a rather general formulation: according to *merits*), he is also hesitant when it comes to a hierarchical evaluation of different occupations and activities. Like Pavel, none of the interviewees elaborates on *how* exactly they envision a just distribution of wages, that is, *who* should receive *how much* from the fruit of *which* labour. One possible explanation for this vagueness can be found in the characteristics of the general formulations of equity (i.e. ‘to each according to work/merit/etc’.). Furthermore, these formulations are considered a ‘form of a social contract’ (Fischer, 2016: 467), so they are generally agreeable and need not be explained further in everyday conversations. At the same time, the formulations remain vague enough to be ‘open to various interpretations and [. . .] implemented differently across situations and individuals’ (Fischer, 2016: 466).

In contrast to the face-to-face interviews, the online survey attempted to pinpoint the respondents’ conception of a fair wage hierarchy with greater precision. They were requested to rank 10 occupations^6^ based on which occupations they believed *should* earn more than others. Interestingly, the results deviate from those of surveys concerning wage hierarchies in other (post-)socialist states, where blue-collar jobs are more valued than white-collar jobs (Duru-Bellat and Tenret, 2012; Gijsberts, 2002; Treiman, 1977). In our sample, the unqualified industrial worker was ranked at the bottom of the list by 49% of respondents and in the bottom three by 72% of respondents. Meanwhile, white-collar workers are clearly at the top of the hierarchy, as 57% of respondents believe that physicians should earn the highest salary. This may reflect either the symbolic and discursive valorisation of this profession in Cuba or the respondents’ orientation to wage hierarchies in the Western world.

### Needs- and equality-based reasoning

In addition to equity-based reasoning, the interviewees also base their arguments on principles of need, and to some extent, on equality principles. Almost all interviewees regard wages, particularly state wages, as insufficient for daily life in Cuba. Most of them frame their reasoning with principles of basic needs, which ‘refer to those that allow a “decent” life in parity with the “normal” standard of living in the person’s society’ (Törnblom and Kazemi, 2015: 26). For example, interviewees believe that wages should be sufficient to live a ‘stable’ (e.g. Ernesto, para. 73) or a ‘decent’ (e.g. Gabriela, para. 37) life, including essentials such as (balanced) nutrition, clothing, and the maintenance of children. This emphasis on basic needs is also reflected in the online survey, where 64% of respondents view ‘what is needed to maintain a minimum standard of living’ as an ‘essential’ criterion for determining wages, while another 32% consider it ‘very important’.

Furthermore, some interviewees discuss needs that may not be considered fundamental but which they nonetheless perceive as important for a decent life. They express these needs using terms such as ‘pleasure’ (e.g. Caridad, para. 68, referring to buying a box of cookies), ‘luxury’ (Manuel, para. 26, referring to university studies), or ‘gratification’ (e.g. Charlie, para. 68). In line with other interviewees, Charlie (30), a financial analyst currently pursuing a part-time university degree in economics, specifically mentions holidays and travel. Charlie portrays himself as content with his life in Cuba, expressing no direct complaints about his salary as a state employee (which he supplements with side jobs in tourism). When asked about the biggest problem of the Cuban realm of work, he initially responds with a generalisation:

Well . . . One thing that most . . . shocks . . . many people, is the salary part. . . . Where . . . (sighs) sometimes mh, it is not enough. . . . [. . .] what I mean is that in general, many people here in Cuba can’t satisfy many of their needs. Or, treat themselves to many things, to the many beauties *(riquesas)* that exist in this country. Eh . . . because, they can’t, that is, eh for . . . they have many situations at home, or, or . . . things to – a lot of things to worry about. Before they can gratify themselves. (Charlie, para. 42)

Charlie’s needs-based argumentation is a carefully formulated critique that suggests that (state) wages in Cuba are insufficient and that Cuban workers in general should earn more. Charlie’s wish expressed later in the interview is that

Cuba ehm, would give . . . would have the opportunity to give people . . . eh, more things. That is to say that with the fruit of your work . . . you can say: Well, this year, I want to spend three days in Varadero. (Charlie, para. 43)

For many contemporary Cubans, a weekend in Varadero (a popular holiday resort) is an unattainable luxury. Charlie believes that if one works, one should be able to afford a vacation in Varadero. This argument can be viewed as an expression of an equality principle, namely that of equal outcomes (for *all workers*). This illustrates how principles of equity and equality can be combined within arguments, a point we will revisit in the conclusion.

### Causes for perceptions of injustice due to equity violations

As exemplified by Alfredo and Pavel’s quotes, the interviewees differ in the extent to which they consider the prevailing wage system to be *unjust.* Yet, many clearly express that they perceive injustices, especially regarding the inverted pyramid resulting from the current system. One (albeit speculative) observation emerging from our analysis is that critiques of perceived injustices that relate to equity principles (i.e. mostly according to qualification and/or effort/performance) fall into two main categories: *blaming structures* and *blaming individuals*. Interviewees who believe that the root causes of injustice primarily lie within the (political and economic) system and its structures criticise the insufficient wage differentiation and low wages in the state sector. For them, this context provides insufficient incentives for work and educational efforts, despite the prestige associated with certain positions. Consequently, they argue that it fosters laziness or unproductivity. Juan, a former surgeon turned taxi driver, sums it up as follows:

[T]he state . . . mh, it doesn’t guarantee you anything. Eh, it’s, it’s like we say a-, eh here in Cuba, I pretend I work, and you pretend you pay me. (Juan, para. 53)

Other interviewees, in turn, see the main problem of the Cuban realm of work in the attitudes and (lacking) effort of the individuals. These interviewees, who consistently highlight their personal achievements and accomplishments, believe that it is the individual lack of work ethic that leads to detrimental effects in the (wage) system and thus produces wage injustice:

There are many people who say that no, eh, the problem is that . . . we are not in a capitalist country. . . . That’s the concept, isn’t it? If . . . you earned according to your work . . . it would be different. And I don’t see it that way. It’s a problem of, as I already told you, education, ethics, values. (Maria, para. 25)

Interestingly, the two categories *blaming structures* and *blaming individuals* appear to correspond with the interviewees’ attitude towards *the political system*.^7^ The analysis reveals that the majority of interviewees who hold a *negative* view of the political system in Cuba identify *structures* as the main cause of problems and injustices in the Cuban realm of work. In contrast, interviewees with a *positive* view of the political system tend to *blame individuals*. In our sample, four out of 19 interviewees (two women and two men) fall into the pattern of *blaming individuals*. According to some studies, different experiences with ‘socialist’ regimes result in generational effects (Ignácz, 2018; for Cuba see, e.g. Krull and Kobayashi, 2009). However, the two identified patterns of views of the political system are not generation-specific, i.e. different generations are found in both patterns.

## Conclusion

**Figure 1.**
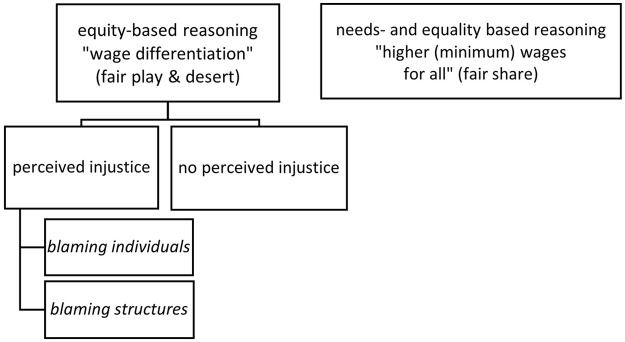
Different reasoning patterns used by the interviewees.

As depicted in Figure 1, we have identified different reasoning patterns among interviewees. Our results confirm the claim made in the justice literature that there is broad support for wage differentiation based on equity principles. At the same time, our data reveal reasoning patterns based on principles of (basic) need and equality (of outcomes). This finding supports the *multi-principle approach of justice*, as well as Fischer’s (2016) claim that ‘various different rules such as [. . .] need and even equality can be reintegrated with equity’ (p. 467). In addition, we outline two different patterns employed by respondents to explain why they perceive equity violations as unjust. We term these patterns ‘blaming structures’ and ‘blaming individuals’. Our observation that equity-based reasoning may be based on people’s attitude towards the country’s political system is an interesting preliminary finding, particularly when considered alongside other studies addressing the issue of ‘justice inferences’ (e.g. Mijs, 2018). In this vein, our findings provide a novel and nuanced perspective on perceptions and reasoning patterns of distributive justice in Cuba. They warrant further exploration both within and beyond the Cuban context.

## Notes

1. During the economic crisis of the 1990s, the US dollar was introduced as a second currency besides the Cuban Peso (CUP); it was replaced in 2004 by a distinct convertible currency, the Convertible Peso (CUC), which prevailed until 2020/2021.

2. We will further address some definitions when discussing the results.

3. These include, for example, doctors, lawyers, architects, engineers, and workers in the hotel and gastronomy business. More detailed information on the samples and data is available upon request.

4. Originally, we planned to use focus groups.

5. It should be noted that the prior completion of the online survey, as well as the fact that some of the participants know each other, may influence research results and could, for example, partially explain similarities in reasoning.

6. The occupations were designated as follows: Physician, manager of a large company, restaurant owner, industrial worker (unqualified), residential landlord, farmer, school teacher, renowned artist, worker in the tourism sector (hotels, transport, guides, . . .), academic professional (engineer, professor, . . .).

7. We draw on interviewees’ direct statements in favour of or against the political system (including statements about the political elite and the government), as well as on accounts of their experiences and behaviour.
